# Nanointerfacial strength between non-collagenous protein and collagen fibrils in antler bone

**DOI:** 10.1098/rsif.2013.0993

**Published:** 2014-03-06

**Authors:** Fei Hang, Himadri S. Gupta, Asa H. Barber

**Affiliations:** 1 Department of Materials, School of Engineering and Materials Science, Queen Mary University of London, Mile End Road, London E1 4NS, UK; 2 National Engineering Research Center for Tissue Restoration and Reconstruction, South China University of Technology, Guangzhou 510640, People's Republic of China; 3 School of Materials Science and Engineering, South China University of Technology, Guangzhou 510640, People's Republic of China

**Keywords:** bone, toughness, nanomechanics

## Abstract

Antler bone displays considerable toughness through the use of a complex nanofibrous structure of mineralized collagen fibrils (MCFs) bound together by non-collagenous proteins (NCPs). While the NCP regions represent a small volume fraction relative to the MCFs, significant surface area is evolved upon failure of the nanointerfaces formed at NCP–collagen fibril boundaries. The mechanical properties of nanointerfaces between the MCFs are investigated directly in this work using an *in situ* atomic force microscopy technique to pull out individual fibrils from the NCP. Results show that the NCP–fibril interfaces in antler bone are weak, which highlights the propensity for interface failure at the nanoscale in antler bone and extensive fibril pullout observed at antler fracture surfaces. The adhesion between fibrils and NCP is additionally suggested as being rate dependent, with increasing interfacial strength and fracture energy observed when pullout velocity decreases.

## 1. Introduction

The failure of interfaces between nanoscale constituents is ubiquitous in tough biological materials, such as bone, shell and their biomimetic equivalents [1–6]. Antler bone is a notable biological material that exhibits considerable toughness by using a fibrous composite structure. The origin of toughness in antler and, indeed, bone material found in other species is contentious, with hierarchical deformation over a range of length scales [7,8], microcracking mechanisms [9,10], heterogeneous failure [11,12], distinctive load transfer between constituents [13] and intrafibrillar plasticity [14] proposed as defining bone toughness. The main structural constituents of antler bone at the nanoscale can be defined as mineralized collagen fibrils (MCFs) bound together by a non-collagenous protein (NCP) region found within the relatively small spaces of 1–2 nm between these collagen fibrils. The NCP region is amorphous and includes a number of proteins, most notably osteopontin [15,16] and proteoglycan tethers between collagen fibrils [17]. These constituents of MCFs and NCPs can be evaluated structurally as a fibrous composite material with a high volume fraction of stiff MCFs acting as a fibre reinforcement within a softer NCP ‘matrix’. The general importance of the collagen fibril unit in macroscopic mechanical properties of biological materials has led to a number of studies examining the mechanical properties of both unmineralized collagen fibrils from various sources [18,19] and MCFs specifically from antler bone tissue [12,20]. However, the NCP region between the collagen fibrils is expected to also be critical in defining the toughness of antler bone. Recent work has indicated this prominence of NCP by showing a deficiency of specific proteins in NCPs causing a loss in strength for tendon [17] and rat bone [21], suggesting that the transfer of stresses between MCFs in antler bone is expected to be critically dependent on the mechanical behaviour of the NCP region. Indeed, classical mechanical analysis of composites consisting of fibres bound together by a polymer matrix highlights the significance of fibre–matrix interfacial adhesion on stress transfer. Composite theory has been extensively exploited in nanocomposite interfacial mechanics, such as efficient stress transfer at carbon nanotube–polymer interfaces [22,23], poor stress transfer in graphene–polymer interfaces [24] and biological nanocomposite structures [25]. Direct mechanical evaluation of the NCP interface region between collagen fibrils in antler bone therefore remains elusive despite corresponding theoretical development and its importance in defining toughness of the antler bone. Direct evaluation of nanoscale interfaces has been achieved using advanced atomic force microscope (AFM) techniques, used to manipulate and remove nanofibres partially embedded within a polymeric matrix material [22,23]. These direct nanofibre pullout measurements give quantitative information on the mechanical behaviour of the interface between the nanofibre and matrix, allowing the evaluation of both strong and weak nanocomposite interfacial mechanics. This paper therefore exploits the direct mechanical testing ability of AFM to evaluate the interfacial properties at collagen fibril–NCP interfaces in antler bone and the role of nanoscale interfaces in antler bone toughness.

## 2. Material and methods

Compact bone samples were extracted from the main beam of antler, after removal of velvet, from a mature red deer (*Cervus elaphus*). All samples were selected from the same compact cortical shell near the antler–pedicle junction. Antler beams of dimensions 3 × 20 × 0.2 mm with the long axis parallel to principal osteonal direction were cut from the bulk material using a water-cooling rotating diamond saw (Struers, Germany) and stored in 70% ethanol solution. Samples were left in Hank's buffered solution overnight to allow full-sample rehydration. Hank's also mitigates mineral loss that may occur in distilled water or physiological saline before pullout testing. Water on the surface of sample was removed by filter paper to avoid interference with scanning electron microscope (SEM) imaging. Hydrated antler bone samples were subsequently fractured perpendicular to their long axis in three-point bending to expose MCFs and immediately transferred to the chamber of an SEM (Quanta 3D, FEI Company, EU/USA) containing a custom-built AFM (Attocube GmbH, Germany). We note that bone samples prepared in such a manner have been shown to remain hydrated in the vacuum chamber of an SEM [26] owing to water being strongly bound to the mineral phase within the timeframe used in this work. This water binding in antler is in contrast to hydrated fibrous collagen without mineral, which can swell [27] and presumably dehydrate significantly if used in the SEM. Antler bone has additionally been shown to be in a dry natural state where higher mechanical property performance is achieved during fighting [28]. The similarity between mechanical testing of mineralized fibrils from antler bone using our experimental set-up within SEM [12] and X-ray techniques that were able to examine the fibrils in hydrated conditions [29] further supports the lack of bound water removal from the antler bone by the SEM partial vacuum.

**Figure 1.**
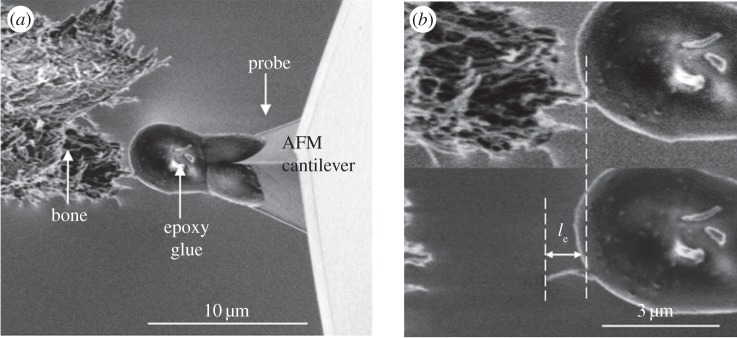
SEM micrographs showing (*a*) an AFM probe containing glue at its apex attached to an individual MCF partially embedded in a fibril bundle at the fracture surface of antler bone and (*b*) higher magnification image showing the pulled out fibril of embedded length *l*_e_.

**Figure 2.**
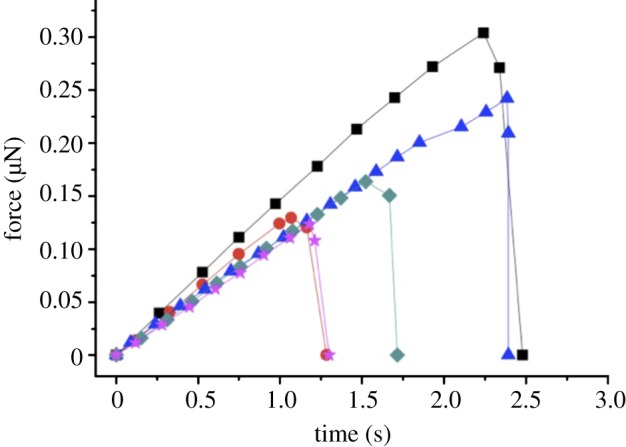
Plot showing the force applied to the partially exposed MCF during pullout against progression time for the pullout experiment. The force increases linearly with progression time until a maximum force *F_p_* is reached, which causes failure of the interface and rapid separation of the MCF from the bulk bone sample. Curves correspond to pullout velocities of 2.30 μm s^−1^ (squares), 1.47 μm s^−1^ (triangles), 1.18 μm s^−1^ (diamonds), 1.03 μm s^−1^ (circles) and 0.61 μm s^−1^ (stars). (Online version in colour.)

MCF pullout from NCP was performed using SEM for imaging with *in situ* AFM providing mechanical testing capability. This AFM–SEM set-up has been previously applied to tensile testing of MCFs [12] and synthetic polymer nanofibres [20], with extensive details of the set-up given previously [20]. Bundles of fibrils at the bone fracture edge were selected with an individual fibril protruding from the centre of the bundle as shown in figure 1. Mechanical testing of the NCP was achieved following a pullout configuration [22]. The AFM probe within the SEM chamber was first translated into a glue droplet (Poxipol, Argentina) contained within the chamber to allow pick-up of glue at the apex of the AFM probe. The AFM probe was then translated towards the free end of the MCF protruding from a bundle as shown in figure 1*a*. This manipulation step was performed within a 10 min time window to ensure that the glue was still liquid during the MCF attachment, after which the glue solidified. The AFM probe was subsequently moved away from the bone surface, which caused an increase in the tensile stress within the fibril and an equal but opposite shear stress within the NCP surrounding the MCF. Fibrils were observed to detach from the bone surface at a critically applied force, measured using an optical interferometer set-up to record the deflection of the AFM cantilever in the AFM system. Any pullout experiments causing glue deformation, owing to insufficient curing of the glue, were discernable using the SEM and discarded. The mechanical properties of the NCP interphase region around the MCFs were calculated by recording the force applied to the MCF by the AFM system. As shown in figure 1*b*, a force *F* is applied to the free end of an MCF with the effective force parallel to the protruding MCF long axis. The force applied to the MCFs shown in figure 2 was calculated from the displacement of the AFM cantilever recorded during the pullout for tests on five different collagen fibrils within the same bone region.

## 3. Results

Pullout of individual MCFs using *in situ* AFM within the chamber of the SEM as shown in figure 1 produced resultant mechanical information during progression of the pullout test as shown in figure 2. The mechanical properties of the NCP interphase region around the MCFs can be calculated by recording the force applied to the MCF by the AFM system. As shown in figure 1, a force *F* is applied at the free end of MCF protruding from the bone material. The pullout force increased linearly with progression time of the experiment (figure 2) until a maximum force, *F_p_*, was reached, which caused failure of the MCF–NCP interface and a rapid drop in the force *F* exerted by the AFM until the MCF was separated from the bone sample. A linear increase in applied force *F* with experimental progression time is generally observed for pullout experiments, for example, when using fibres with nano- and micro-sized diameters [22,30], indicating that the MCFs in this work pull out as opposed to fracture within the bulk bone material and subsequent pullout. We also note that the increase in force shown in figure 2 to a maximum value indicates that a critical stress is required to fail a coherent interface, whereas a prefailed interface, potentially owing to the preparation of the fracture surface, would require the applied force to only overcome a frictional adhesion. This frictional adhesion would be highlighted as a force plateau with experiment time in figure 2 and, as this is not observed, shows that sample preparation did not prefail MCF–NCP interfaces.

The strength of the interface between the MCF and surrounding NCP is characterized by the interfacial shear strength (*τ*_*i*_) and is calculated from the maximum force applied to the exposed MCF to cause pullout from the surrounding NCP using the force balance3.1
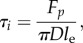
where *D* is the fibril diameter and *l*_e_ is the length of fibril embedded within the bone. Equation (3.1) assumes the shear stress generated at the MCF–NCP interface during pullout is uniformly distributed along the fibril embedded length. As with previous pullout experiments [22,30,31], MCF end effects owing to bonding between the end of the MCF and NCP are relatively minor and ignored. The evaluation of the interfacial shear strength at the MCF–NCP interface thus requires accurate determination of fibril diameter *D* and embedded length *l*_e_. SEM was used to measure the diameter and length of the MCF before and after pullout testing. The diameter of the MCF fibrils, measured at five equidistant points along the MCF free length, was reasonably constant with an average *D* = 115 ± 33 nm. No fibril tapering was observed from SEM measurements, suggesting that a fibril fragment was being pulled out as opposed to a fibril end. The length of the MCF observed using SEM after pullout consists of the MCF free length before pullout and the embedded length *l*_e_. Thus, subtraction of the MCF length before pullout from the MCF length after pullout provides *l*_e_. SEM imaging of the MCF free lengths before and after pullout using pixel analysis (ImageJ, NIH, USA) gave a range of *l*_e_ values from 510 to 880 nm. Using the pullout forces of this work, the strain in the free length of the MCF during pullout testing is less than 1%, calculated from the force applied to the MCF and the elastic modulus of the MCF [12], indicating negligible fibril strain contributions.

Solving equation (3.1) using the maximum pullout force results in figure 2 gives a calculated MCF–NCP interfacial shear strength *τ*_*i*_ = 0.65 ± 0.15 MPa. This shear strength for nanoscale interfaces found in bone is lower than values recorded from pullout of engineering fibres from conventional fibre-reinforced polymer composites [30,31]. Specifically, engineering composites are often optimized for effective stress transfer between the reinforcing fibres and require relatively high *τ*_*i*_ values of up to approximately 50 MPa [31]. The MCF fibres in this work therefore exhibit low interfacial shear strength. We note that the forces used to cause fibril pullout will also cause a tensile stress in the MCF. However, the maximum tensile stresses generated in the MCF during pullout, which can be simply found by dividing the maximum forces *F_p_* by the fibril cross-sectional areas from data in table 1, are an order of magnitude lower than the tensile strength of the fibrils [12]. We can therefore confirm that our experiments caused pullout of the fibril lengths listed in table 1 in preference to MCF tensile failure. As no evidence for fibril failure is observed in the loading curve in figure 2, the short fibril must have been produced from longer fibril lengths that potentially fragmented during preparation of the sample.

**Table 1.** Data showing the fibril geometry, the resultant pullout behaviour including the work done and calculated interfacial shear strength, as well as interfacial fracture energy when pulling out MCFs from NCPs at various velocities.

*l*_e_ (nm)	diameter (nm)	work (×10^−14^ J)	*τ*_*I*_(MPa)	*γ*_*a*_(J m^−2^)	pullout velocity (*μ*m s^−1^)
700 ± 35	168 ± 4	7.89 ± 0.12	0.61 ± 0.06	0.21 ± 0.02	1.47
510 ± 26	102 ± 3	2.96 ± 0.04	0.71 ± 0.07	0.18 ± 0.02	1.18
740 ± 37	110 ± 3	7.00 ± 0.10	0.74 ± 0.07	0.27 ± 0.03	0.61
880 ± 44	121 ± 3	6.03 ± 0.11	0.41 ± 0.04	0.18 ± 0.02	2.30
540 ± 27	81 ± 3	2.93 ± 0.07	0.79 ± 0.08	0.21 ± 0.03	1.03

**Figure 3.**
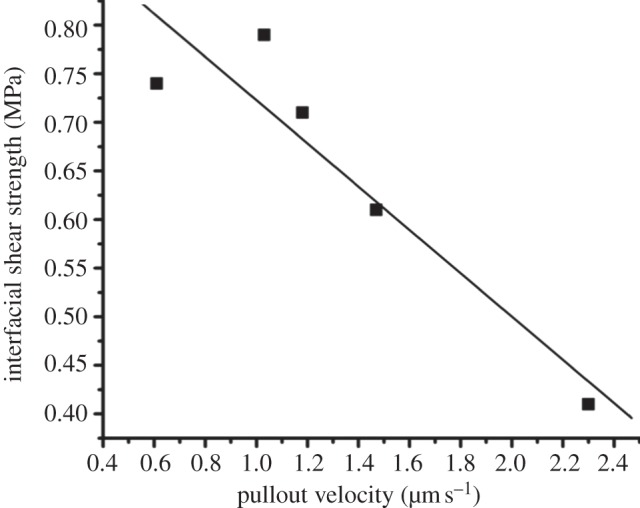
Plot of the variation in the MCF–NCP interfacial shear strength, calculated using equation (3.1), with pullout velocity. A simple linear trend line highlights the increase in interfacial shear strength with decreasing pullout velocity.

Previous literature for both nano [22–24] and engineering [30,31] composites highlights the use of weak interfaces to deflect cracks propagating through the material using a variety of mechanisms, which enhance composite toughness by increasing the resultant composite fracture surface area. Therefore, the observed weak MCF–NCP interfaces in antler bone are conducive to toughness as cracks propagating through bone will be deflected at the weak interfaces between the MCFs and surrounding NCP, as observed at fracture surfaces in figure 1. The vast area available at these nanoscale interfaces in bone will be a considerable energy-absorbing process. To explore the interfacial failure mechanism further, various testing rates were examined to understand the dynamic behaviour of the bone material. The time-dependent failure of the MCF–NCP interface can be evaluated by controlling the displacement of the pullout experiment. Indeed, the pullout process should be comparable to the physiological loading rate experienced in antler bone. Figure 3 shows the variation in the interfacial shear strength over a range of pullout velocities. We note that the physiological loading velocity for antler bone is approximately 1 μm s^−1^ as derived from strains rates of approximately 1.6 s^−1^ reported in previous literature [32] and from the pullout lengths used in this work, and is within the range of pullout velocities examined in figure 3. The rate-dependent mechanical behaviour at the MCF–NCP interface highlights a clear increase in the interfacial shear strength with decreasing pullout velocity in figure 3. Thus, the MCF–NCP interface is shown to be both relatively weak but exhibiting rate-dependent mechanical behaviour using the experimental pullout testing configuration.

## 4. Discussion

The nature of the adhesion between the MCFs and NCP at bone interfaces can be assessed using energy-based criteria [33]. To understand this adhesion within bone at the nanoscale, the work done *W* to pull out the collagen fibrils from the NCP can be simply calculated from the area under a force–displacement plot. The force increases in a relatively linear manner with sample progression, and thus displacement, in figure 3 that allows an estimation of *W* from the maximum pullout force *F_p_* applied to displace the MCF by its embedded length *l*_e_, thus4.1
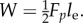


**Figure 4.**
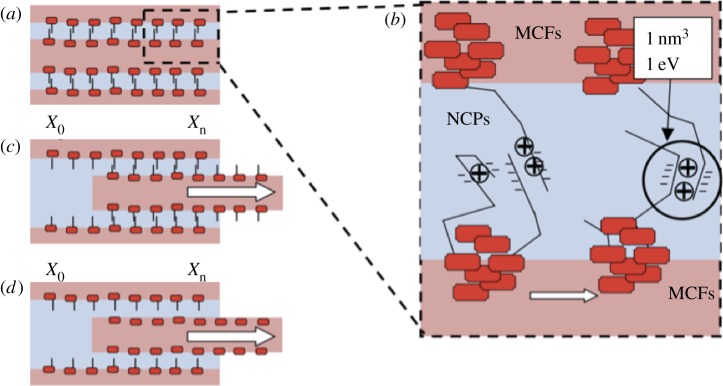
Schematic of the sacrificial ionic bonding system in NCPs region showing MCFs (dark) with squares indicating mineral crystals bonding with extrafibrillar NCP region (light). Each short line in the NCP layer represents an activation volume, previous defined as an activation energy of 1 eV for plasticity acting over approx. 1 nm^3^ [33]. (*a,b*) The negatively charged molecules bonded by positively charged divalent calcium ions. The arrows indicate the movement of the MCFs causing shear in the NCPs layer during individual MCF pullout. The two interface-bonding boundary conditions indicate either (*c*) all ionic bonds reform reversibly during the process of MCF pullout or (*d*) no ionic bonds reform in the pullout process. (Online version in colour.)

This work required to pull out the fibril causes deformation and resultant failure of the NCP, and therefore requires further description of the NCP in order to understand the observed pullout behaviour. At molecular length scales, the NCP material consists of proteins, including osteopontin [15], proteoglycans [34,35] and fetuin A [36], that are anchored to the mineral sites on the MCFs and form an ionic network. The ionic bonds existing in the thin layer of extrafibrillar NCPs bridge between negatively charged protein molecules and divalent calcium ions, providing a molecular ‘glue’ to bind the MCFs together as shown in figure 4. These negatively charged protein molecules are bound to the mineral regions of the MCF [37,38] so that a network connectivity between the fibrils occurs in figure 4. The work done to pull out the MCF from surrounding NCP will cause failure of these sacrificial ionic bonds in the NCP glue. However, a number of works have shown how the interfacial region can reform [37,39,40], indicating that the work of pullout may cause failure of the sacrificial ionic bonds many times in the NCP before complete MCF separation from the bone bulk. A simple description to show the increase in work needed to separate the fibrils from one another in during pullout can be made using the experimental data in table 1. A crack propagates along the interface will cause separation of the molecular network shown in figure 4. The calculated work done during pullout *W* is related to the interfacial fracture energy *γ*_*a*_, with units of J m^−2^, using4.2
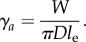

**Figure 5.**
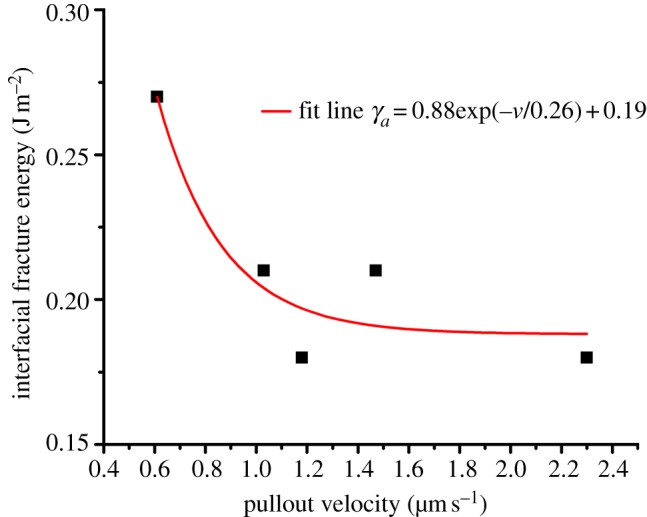
Plot of the interfacial fracture energy during pullout of individual MCFs from the NCP at various velocities. (Online version in colour.)

Resultant *γ*_*a*_ values can be calculated using equation (4.2) and are plotted in figure 5 with pullout velocity. The fracture energy expectedly increases with decreasing pullout velocity in a similar manner to the interfacial strength in figure 3. The interfacial fracture energy for pullout of MCFs from NCP is further shown in table 1. The magnitude of this interfacial fracture energy derived from pullout is comparable to values for synthetic soft adhesive materials [41,42]. This comparison indicates that the NCP region is indeed soft as supported by previous work recording a low Dalton number [15,16] for osteopontin, a major component of NCP. However, the interfacial fracture energy suggests an increase with decreasing loading rate and is opposite to the response of these synthetic soft adhesive materials. We therefore believe that bond reforming within the NCP is responsible for the increase in the interfacial fracture energy with lower pullout velocity and speculate that the bond reforming is controlled by calcium ion mobility within the NCP region. The speed of calcium ion migration to new bond reforming sites, which is defined by the permeability of NCP, is critical to the reforming process. More rapid pullout testing would allow insufficient time for the calcium ions to transport sufficiently to bond reforming sites, which leads to a lower fracture energy. Slower pullout velocities will therefore provide more time for ionic bonds to reform, resulting in higher fracture energy. These calculations warrant comparison with other materials tested dynamically. Macroscopic elk antler samples have been mechanically tested in compression and have exhibited an increase in their compressive strength with strain rate [43]. However, these previous tests use strain rates over a larger range than in this work that suggests a nonlinear mechanical dependence. Compressive testing of large samples is also difficult to directly compare to the shear testing of antler bone's material properties at nanometre length scales that exclude structural hierarchy effects. Self-healing polymer gels mimicking mussel thread cuticle mechanically tested in shear present a more relevant system and critically exhibit increasing mechanical properties with decreasing strain rate [44], suggesting that the increased mechanical properties with decreasing strain rate owing to ionic bonds reforming may be a general design feature in dynamically loaded biological systems. Furthermore, the resultant MCF–NCP interfacial strength is important in defining the toughness of antler bone. The relatively low fracture energy recorded from pullout at physiological loading rates is critical in promoting delocalization of damage as cracks propagating through antler bone will be easily deflected at the weak interfaces between MCFs. This crack deflection at the nanoscale is analogous to extensive crack deflection found at larger length scales in bone material [9,10]. Indeed, the low interfacial shear strength values recorded in this work suggest that considerable interfacial fracture area will be evolved during antler bone fracture in order to be considered as a major energy-absorbing process. The interface is additionally expected to transfer stresses to MCFs in order to promote fibril failure. The failure of MCFs is a significant energy-absorbing process relative to MCF pullout, with approximately 10^−8^ J required to fail an individual MCF [12], whereas only approximately 10^−14^ J (as shown in table 1) is required to pull out submicrometre MCF lengths from the NCP. The interfacial shear stresses cause a corresponding building of tensile stresses in the MCF, which increase as the aspect ratio of the fibrous unit increases [22]. Thus, despite the low shear strength at the MCF–NCP interface, the relatively high aspect ratio MCFs will promote tensile stresses potentially sufficient to fail the fibril. The rate-dependent interfacial shear strength is more difficult to interpret as a maximum interfacial shear strength is not observed around physiological loading rates, indicating that the interface is not optimized as an energy-absorbing process during antler bone loading.

## 5. Conclusion

Individual MCFs were pulled out of bone material and the resultant mechanical properties of the NCP interface with fibrils were measured. Specifically, the MCF–NCP interfacial adhesion was found to be relatively weak but increased as the pullout rate decreased. While this interfacial adhesion is low compared with engineered fibre composites, the high fibril aspect ratio and low fibril diameter ensure that considerable interfacial area is available to fail, thus providing a number of pathways for nanoscale cracking. Weak interfaces are associated with delocalization of failure commonly observed at larger length scales in bone materials [9–11] with the results of this paper quantitatively highlight the propensity for interfacial failure at the nanoscale.

## References

[1] Li XD Chang WC Chao YJ Wang RZ Chang M 2004 Nanoscale structural and mechanical characterization of a natural nanocomposite material: the shell of red abalone. Nano Lett. 4, 613–617. (10.1021/nl049962k)

[2] Yao HM Dao M Imholt T Huang J Wheeler K Bonilla A Suresh S Ortiz C 2010 Protection mechanisms of the iron-plated armor of a deep-sea hydrothermal vent gastropod. Proc. Natl Acad. Sci. USA 107, 987–992. (10.1073/pnas.0912988107) 20133823 PMC2808221

[3] Chai H Lee JJW Constantino PJ Lucas PW Lawn BR 2009 Remarkable resilience of teeth. Proc. Natl Acad. Sci. USA 106, 7289–7293. (10.1073/pnas.0902466106) 19365079 PMC2678632

[4] Bonderer LJ Studart AR Gauckler LJ 2008 Bioinspired design and assembly of platelet reinforced polymer films. Science 319, 1069–1073. (10.1126/science.1148726) 18292337

[5] Podsiadlo P 2007 Ultrastrong and stiff layered polymer nanocomposites. Science 318, 80–83. (10.1126/science.1143176) 17916728

[6] Munch E Launey ME Alsem DH Saiz E Tomsia AP Ritchie RO 2008 Tough, bio-inspired hybrid materials. Science 322, 1516–1520. (10.1126/science.1164865) 19056979

[7] Rho JY Kuhn-Spearing L Zioupos P 1998 Mechanical properties and the hierarchical structure of bone. Med. Eng. Phys. 20, 92–102. (10.1016/S1350-4533(98)00007-1) 9679227

[8] Currey JD 2005 Materials science—hierarchies in biomineral structures. Science 309, 253–254. (10.1126/science.1113954) 16002605

[9] Peterlik H Roschger P Klaushofer K Fratzl P 2006 From brittle to ductile fracture of bone. Nat. Mater. 5, 52–55. (10.1038/nmat1545) 16341218

[10] Koester KJ Ager JW Ritchie RO 2008 The true toughness of human cortical bone measured with realistically short cracks. Nat. Mater. 7, 672–677. (10.1038/nmat2221) 18587403

[11] Tai K Dao M Suresh S Palazoglu A Ortiz C 2007 Nanoscale heterogeneity promotes energy dissipation in bone. Nat. Mater. 6, 454–462. (10.1038/nmat1911) 17515917

[12] Hang F Barber AH 2011 Nano-mechanical properties of individual mineralized collagen fibrils from bone tissue. J. R. Soc. Interface 8, 500–505. (10.1098/rsif.2010.0413) 20961895 PMC3061121

[13] Gupta HS Seto J Wagermaier W Zaslansky P Boesecke P Fratzl P 2006 Cooperative deformation of mineral and collagen in bone at the nanoscale. Proc. Natl Acad. Sci. USA 103, 17 741–17 746. (10.1073/pnas.0604237103) PMC1635545 17095608

[14] Gupta HS Krauss S Kerschnitzki M Karunaratne A Dunlop JWC Barber AH Boesecke P Funari SS Fratzl P 2013 Intrafibrillar plasticity through mineral/collagen sliding is the dominant mechanism for the extreme toughness of antler bone. J. Mech. Behav. Biomed. Mater. 28, 366–382. (10.1016/j.jmbbm.2013.03.020) 23707600

[15] Sodek J Ganss B McKee MD 2000 Osteopontin. Crit. Rev. Oral Biol. Med. 11, 279–303. (10.1177/10454411000110030101) 11021631

[16] Nanci A 1999 Content and distribution of noncollagenous matrix proteins in bone and cementum: relationship to speed of formation and collagen packing density. J. Struct. Biol. 126, 256–269. (10.1006/jsbi.1999.4137) 10441531

[17] Cribb AM Scott JE 1995 Tendon response to tensile-stress—an ultrastructural investigation of collagen-proteoglycan interactions in stressed tendon. J. Anat. 187, 423–428. 7592005 PMC1167437

[18] van der Rijt JAJ van der Werf KO Bennink ML Dijkstra PJ Feijen J 2006 Micromechanical testing of individual collagen fibrils. Macromol. Biosci. 6, 697–702. (10.1002/mabi.200600063) 16967482

[19] Shen ZL Dodge MR Kahn H Ballarini R Eppell SJ 2008 Stress–strain experiments on individual collagen fibrils. Biophys. J. 95, 3956–3963. (10.1529/biophysj.107.124602) 18641067 PMC2553131

[20] Hang F 2011 *In situ* tensile testing of nanofibers by combining atomic force microscopy and scanning electron microscopy. Nanotechnology 22, 365708 (10.1088/0957-4484/22/36/365708) 21844643

[21] Arteaga-Solis E Sui-Ateaga L Kim M Schaffler MB Jepsen KJ Pleshko N Ramirez F 2011 Material and mechanical properties of bones deficient for fibrillin-1 or fibrillin-2 microfibrils. Matrix Biol. 30, 188–194. (10.1016/j.matbio.2011.03.004) 21440062 PMC3097426

[22] Barber AH Cohen SR Eitan A Schadler LS Wagner HD 2006 Fracture transitions at a carbon-nanotube/polymer interface. Adv. Mater. 18, 83–87. (10.1002/adma.200501033)

[23] Barber AH Cohen SR Kenig S Wagner HD 2004 Interfacial fracture energy measurements for multi-walled carbon nanotubes pulled from a polymer matrix. Compos. Sci. Technol. 64, 2283–2289. (10.1016/j.compscitech.2004.01.023)

[24] Gong L Kinloch IA Young RJ Riaz I Jalil R Novoselov KS 2010 Interfacial stress transfer in a graphene monolayer nanocomposite. Adv. Mater. 22, 2694–2697. (10.1002/adma.200904264) 20473982

[25] Ji B Gao H 2010 Mechanical principles of biological nanocomposites. Ann. Rev. Mater. Res. 40, 77–100. (10.1146/annurev-matsci-070909-104424)

[26] Jimenez-Palomar I Shipov A Shahar R Barber AH 2012 Influence of SEM vacuum on bone micromechanics using *in situ* AFM. J. Mech. Behav. Biomed. Mater. 5, 149–155. (10.1016/j.jmbbm.2011.08.018) 22100089

[27] Shen ZL Kahn H Ballarini R Eppell SJ 2011 Viscoelastic properties of isolated collagen fibrils. Biophys. J. 100, 3008–3015. (10.1016/j.bpj.2011.04.052) 21689535 PMC3123930

[28] Currey JD Landete-Castillejos T Estevez J Ceacero F Olguin A Garcia A Gallego L 2009 The mechanical properties of red deer antler bone when used in fighting. J. Exp. Biol. 212, 3985–3993. (10.1242/jeb.032292) 19946076

[29] Gupta HS Wagermaier W Zickler GA Aroush DRB Funari SS Roschger P Wagner HD Fratzl P 2005 Nanoscale deformation mechanisms in bone. Nano Lett. 5, 2108–2111. (10.1021/nl051584b) 16218747

[30] Hsueh CH 1991 Interfacial debonding and fiber pull-out stresses of fiber-reinforced composites. 6. Interpretation of fiber pull-out curves. Mater. Sci. Eng. A 149, 11–18. (10.1016/0921-5093(91)90781-H)

[31] Barber AH Wiesel E Wagner HD 2002 Crack deflection at a crystalline junction. Compos. Sci. Technol. 62, 1957–1964. (10.1016/S0266-3538(02)00112-4)

[32] Currey JD 1989 Strain rate dependence of the mechanical properties of reindeer antler and the cumulative damage model of bone-fracture. J. Biomech. 22, 469–475. (10.1016/0021-9290(89)90207-8) 2777821

[33] Gupta HS Fratzl P Kerschnitzki M Benecke G Wagermaier W Kirchner HOK 2007 Evidence for an elementary process in bone plasticity with an activation enthalpy of 1 eV. J. R. Soc. Interface 4, 277–282. (10.1098/rsif.2006.0172) 17251154 PMC2220070

[34] Scott JE 1988 Proteoglycan fibrillar collagen interactions. Biochem. J. 252, 313–323. 3046606 10.1042/bj2520313 PMC1149146

[35] Scott JE Haigh M 1985 Proteoglycan-type I collagen fibril interactions in bone and non-calcifying connective tissues. Biosci. Rep. 5, 71–81. (10.1007/bf01117443) 3986311

[36] Heiss A DuChesne A Denecke B Grotzinger J Yamamoto K Renne T Jahnen-Dechent W 2003 Structural basis of calcification inhibition by alpha(2)-HS glycoprotein/fetuin-A—formation of colloidal calciprotein particles. J. Biol. Chem. 278, 13 333–13 341. (10.1074/jbc.M210868200) 12556469

[37] Hartmann MA Fratzl P 2009 Sacrificial ionic bonds need to be randomly distributed to provide shear deformability. Nano Lett. 9, 3603–3607. (10.1021/nl901816s) 19725552 PMC2762307

[38] Salih E Ashkar S Gerstenfeld LC Glimcher MJ 1997 Identification of the phosphorylated sites of metabolically P-32-labeled osteopontin from cultured chicken osteoblasts. J. Biol. Chem. 272, 13 966–13 973. (10.1074/jbc.272.21.13966) 9153260

[39] Currey J 2001 Biomaterials—sacrificial bonds heal bone. Nature 414, 699 (10.1038/414699a) 11742376

[40] Fantner GE 2005 Sacrificial bonds and hidden length dissipate energy as mineralized fibrils separate during bone fracture. Nat. Mater. 4, 612–616. (10.1038/nmat1428) 16025123

[41] Bhuyan S Tanguy F Martina D Lindner A Ciccotti M Creton C 2013 Crack propagation at the interface between soft adhesives and model surfaces studies with a sticky wedge test. Soft Matter 9, 6515–6524. (10.1039/c3sm27919g)

[42] Léger L Creton C 2008 Adhesion mechanisms at soft polymer interfaces. Phil. Trans. R. Soc. A 366, 1425–1442. (10.1098/rsta.2007.2166) 18156130

[43] Kulin RM Chen P-Y Jiang FC Vecchio KS 2011 A study of the dynamic compressive behavior of elk antler. Mater. Sci. Eng. C 31, 1030–1041. (10.1016/j.msec.2011.03.002)

[44] Holten-Anderson N Harrington MJ Birkedal H Lee BP Messersmith PB Lee K-YC Waite JH 2011 pH-induced metal-ligand cross-links inspired by mussel yield self-healing polymer networks with near-covalent elastic moduli. Proc. Natl Acad. Sci. USA 15, 2651–2655. (10.1073/pnas.1015862108) PMC3041094 21278337

